# A microfluidic platform for cultivating ovarian cancer spheroids and testing their responses to chemotherapies

**DOI:** 10.1038/s41378-020-00201-6

**Published:** 2020-10-19

**Authors:** Neda Dadgar, Alan M. Gonzalez-Suarez, Pouria Fattahi, Xiaonan Hou, John S. Weroha, Alexandre Gaspar-Maia, Gulnaz Stybayeva, Alexander Revzin

**Affiliations:** 1 Department of Physiology and Biomedical Engineering, Mayo Clinic, Rochester, MN 55905 USA; 2 Department of Medical Oncology, Mayo Clinic, Rochester, MN 55905 USA; 3 Department of Laboratory Medicine and Pathology, Mayo Clinic, Rochester, MN 55905 USA

**Keywords:** Microfluidics, Environmental, health and safety issues

## Abstract

There is increasing interest in utilizing in vitro cultures as patient avatars to develop personalized treatment for cancer. Typical cultures utilize Matrigel-coated plates and media to promote the proliferation of cancer cells as spheroids or tumor explants. However, standard culture conditions operate in large volumes and require a high concentration of cancer cells to initiate this process. Other limitations include variability in the ability to successfully establish a stable line and inconsistency in the dimensions of these microcancers for in vivo drug response measurements. This paper explored the utility of microfluidics in the cultivation of cancer cell spheroids. Six patient-derived xenograft (PDX) tumors of high-grade serous ovarian cancer were used as the source material to demonstrate that viability and epithelial marker expression in the microfluidic cultures was superior to that of Matrigel or large volume 3D cultures. To further demonstrate the potential for miniaturization and multiplexing, we fabricated multichamber microfluidic devices with integrated microvalves to enable serial seeding of several chambers followed by parallel testing of several drug concentrations. These valve-enabled microfluidic devices permitted the formation of spheroids and testing of seven drug concentrations with as few as 100,000 cancer cells per device. Overall, we demonstrate the feasibility of maintaining difficul-to-culture primary cancer cells and testing drugs in a microfluidic device. This microfluidic platform may be ideal for drug testing and personalized therapy when tumor material is limited, such as following the acquisition of biopsy specimens obtained by fine-needle aspiration.

## Microfluidics: A novel platform for ovarian cancer cultures

Researchers in the United States have developed a microfluidic system for culturing ovarian cancer organoids. The use of microfluidics makes it possible to create cultures starting from a smaller number of cells than existing approaches. The team compared the new approach to standard techniques and found that the resulting cultures were superior in terms of viability, proliferation, and phenotype expression. Building on this framework, the researchers developed an 8-chamber microfluidic device which could be seeded serially and then used for parallel testing of different drug concentrations. The device required with fewer than 100,000 cells, which is insufficient to test multiple conditions using existing approaches. The ability to work reliably with small cells numbers makes this microfluidic platform well-suited for personalized drug testing when the sample material is limited.

## Introduction

Ovarian cancer (OC) remains one of the most lethal gynecologic cancers, with 22,440 new cases and 14,080 deaths annually^1^. The high-grade serous histologic subtype is the most common type of OC^2^. Most patients are diagnosed with advanced-stage disease that has already spread within the pelvis and abdomen, and ~75% of patients relapse after surgery and adjuvant chemotherapy^3^. The need for better therapeutic strategies is critical for the treatment of OC. A barrier to the study of novel therapies for OC has been the paucity of clinically relevant models. This spurred the development of primary patient-derived xenograft (PDX) OC models that recapitulate patient disease in terms of histologic, genomic, transcriptomic, and therapeutic heterogeneity^4–6^. Although the responses of PDX models to standard chemotherapy, such as carboplatin and paclitaxel, parallel those observed in patients^6^, PDX experiments require skilled animal technicians, higher costs, and longer timelines than in vitro studies. This motivates the need for in vitro culture systems that may be used to maintain the phenotype of primary tumors over days or weeks while testing their drug responsiveness and resistance^7,8^.

3D cancer cultures (organoids or spheroids) represent a tool for maintaining patient-specific tumors in vitro. First described in the context of pancreatic cancer^9,10^, spheroid cultures have since been successfully developed for liver, lung, esophageal and other cancers^10–12^. The development of spheroid cultures of serous OC has also been reported in recent studies, showing the genomic and functional similarities between primary tumor tissue and matched OC^13,14^. The emergence of spheroids as OC patient surrogates will be further boosted by the availability of culture systems that can use small amounts of tumor tissue obtained from image-guided needle biopsies for testing anticancer drugs.

There are several techniques used for forming 3D spheroids, such as suspension cultures (on top of solid extracellular matrix), hanging drop, the use of spinner flasks or round-bottom ultralow attachment plates, and magnetic levitation^15–17^. Such techniques rely on the absence of an attachment surface and the construction of an environment where cell–cell interactions are increased to enable the successful formation of compact spheroids. Spheroid formation methods have been translated to microfluidic devices^18^, which allow the utilization of minimal amounts of tissue and reagents and have been gaining popularity as cancer cell culture platforms that allow for the integration of different tumor environment components, such as the stroma^19–22^ and the immune system^23–26^.

A number of microfluidic strategies aimed at the formation of cellular spheroids have been reported in the literature^18^. Droplet-generation devices have been utilized to create capsules with cancer cells embedded in extracellular matrix^27,28^. However, such encapsulation strategies may be challenging to adapt to scenarios where cell availability is limited. The hanging drop method has been integrated with microfluidic devices and has been combined with other microfluidic modules such as concentration gradient generators, and it has been used as a drug testing platform^29,30^. However, these microfluidic hanging drop devices rely on the integration of porous membranes with fluid channels, which represents a fabrication challenge. Hydrodynamic traps have also been used to confine cells in U-shaped structures to allow spheroid formation^31,32^; however, the nature of cell trapping requires the input of a large number of cells to account for inefficient trapping. The use of microwells is a simple method that relies on sedimentation of cells into confined spaces (~300 µm) to allow spheroid formation^33–35^. Microwell-containing microfluidic devices are simple to fabricate and may be efficiently seeded with a small number of input cells. Therefore, we chose to employ these devices in our study.

In this paper, we focused on characterizing the viability, proliferation and phenotype maintenance of spheroids established from six different PDX models of OC. Comparison of standard and microfluidic cancer cultures revealed the latter to be superior in terms of viability and expression of epithelial cancer phenotype. In addition, we demonstrated the development of an 8-chamber microfluidic cancer culture platform that includes automated microvalves and allows for orthogonal perfusion of chambers: serial perfusion for seeding and parallel perfusion for testing different drug concentrations.

## Results and discussion

The overall objective of this paper was to establish the suitability of microfluidic devices for the cultivation of OC spheroids. Throughout this study, we utilized six OC PDX lines—039, 063, 592, 704, 757, and 938. All but one of the lines (PH592) was of high-grade serous carcinoma histotype—the most common and lethal type of OC. PH592 belongs to the rarer, malignant mixed mesodermal tumor (MMMT) type, which is characterized by the presence of both epithelial and mesenchymal cancer cells^36^.

**Fig. 1:**
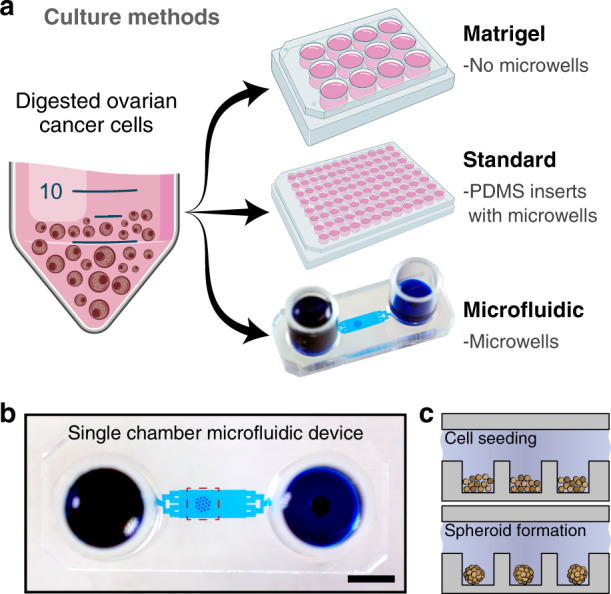
Comparison of OC cultivation with three different methods. **a** Culture methods for OC spheroids. Matrigel cultures established in 6-well plates coated with 100% Matrigel containing no microwells. Both standard and microfluidic cultures contained microwells of the same dimensions (250 μm diameter and 300 μm in depth) for spheroid formation. **b** An image of a microfluidic chamber containing an array of 19 microwells for spheroid formation. The array of spheroids was connected to media reservoirs via a transport channel. The dotted square shows the location of the microwells. Scale bar = 5 mm. **c** Schematic depicting seeding of cells and spheroid formation. The PDMS surface inside the device is functionalized by treatment with Pluronic F-127 to minimize cell-surface interactions and promote cell–cell interactions

We directly compared OC spheroid cultures using three different culture methods (see Fig. 1a). (1) *Matrigel*—6-well plates coated with Matrigel (not containing microwells); (2) *Standard*—96-well plates with PDMS inserts containing microwells; and (3) *Microfluidic*—microfluidic devices composed of PDMS and containing microwells. The same medium was used for all three culture systems, and the medium was changed every 24 h. Upon characterizing the maintenance of spheroids in a simpler microfluidic device, we fabricated a valve-enabled multichamber microfluidic device for parallel testing of several drug concentrations.

### Formation of OC spheroids in microfluidic devices

**Fig. 2:**
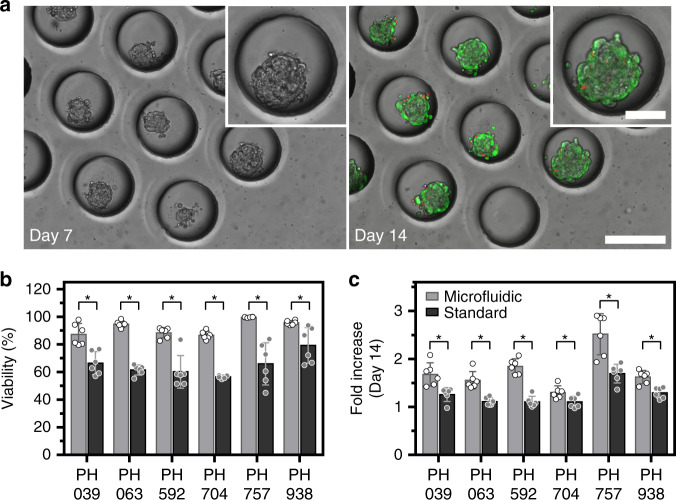
Cultivation of OC spheroids in a single chamber microfluidic device. **a** Micrographs showing microwell areas from a microfluidic device with spheroids after 7 and 14 days of culture (scale bar = 250 µm). The insert shows a zoomed-in image of a single microwell/spheroid (scale bar = 100 µm). The right image shows live/dead staining at day 14 (live (green), dead (red fluorescence)). **b** Viability of spheroids from the six PDX lines after 14 days of culture were tested in this study to compare microfluidic (gray bars) versus standard (black bars) culture methods. Live/dead staining was used for quantification (*p* < 0.05, *n* = 6). **c** Increase in spheroid diameter was observed after 14 days of culture (*p* < 0.05, *n* = 6)

Cell characterization experiments were carried out in simple microfluidic devices of the type shown in Fig. 1b. These devices were functionalized by treatment with Pluronic F-127 to prevent cell-surface interactions and promote cell–cell aggregation. This aggregation occurred in the 48–72 h after cell seeding (Fig. 1c), resulting in the formation of compact spheroids. Fig. 2a shows images of spheroid arrays at different time points of microfluidic culture. As seen in these images, cancer cell clusters were of a reproducible size and shape. In addition, live/dead staining revealed that OC spheroids remained highly viable after 14 days of culture (see Fig. 2b).

#### Is there a benefit to forming spheroids in microfluidic devices vs. standard 3D cultures?

To address this question, we fabricated PDMS inserts containing arrays of microwells with the same dimensions (250 µm diameter, 300 µm depth, and 125-µm edge-to-edge distance) as the microwells used in microfluidic devices. These PDMS inserts were placed into wells of a 96-well plate and then were treated with Pluronic F-127 to promote spheroid formation. Cancer cells from six different OC PDX lines were seeded into microfluidic devices of the type shown in Fig. 1b and into 96-well plates with PDMS inserts to compare spheroid growth and cell viability. Fig. 2b, c highlights the fact that after 14 days of culture, viability was significantly better (*p* < 0.05) in microfluidic devices than it was in standard 3D cultures for all 6 OC PDX lines tested. It is worth noting that the same media was used for both culture formats.

Our lab has previously demonstrated that small volume microfluidic cultures elicit an improved phenotype in a range of cell types, including cancer cells^37^. These improvements were connected to autocrine signaling, which was more pronounced in small volume microfluidic cultures. We speculate that similar effects contribute to the improved OC spheroid growth observed in Fig. 2c; however, specific autocrine signals contributing to this behavior remain to be elucidated.

#### How do microfluidic cultures compare to Matrigel-based spheroid cultures?

**Fig. 3:**
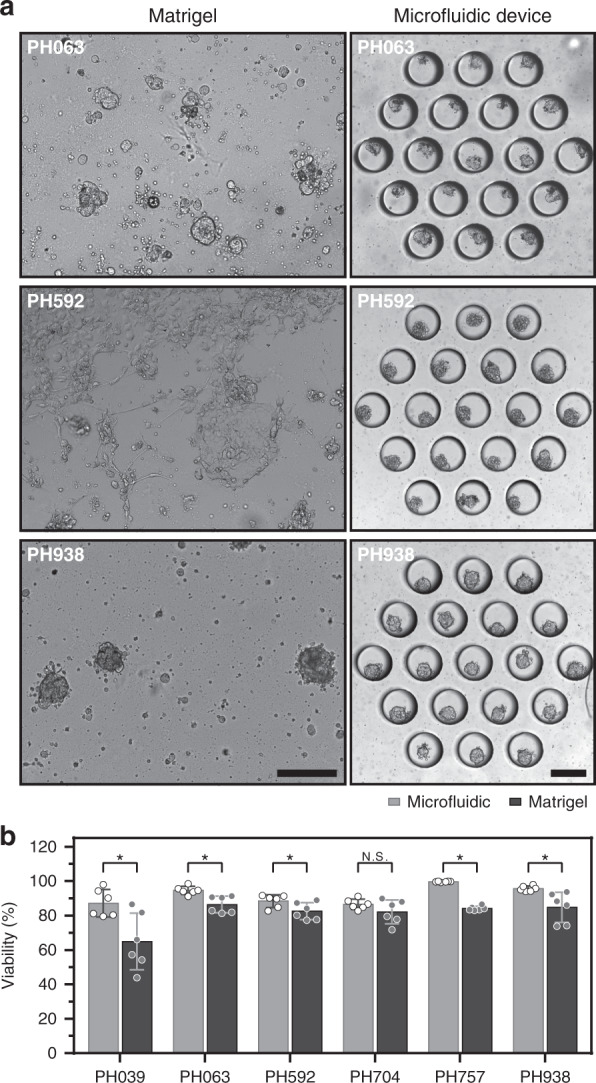
Comparison of spheroid cultures on Matrigel and in microfluidic devices. **a** Micrographs showing cells from three different PDX lines cultured on Matrigel and in microfluidic microwells. Note the difference the uniform spheroid formation in microfluidic microwells with the variable spheroid sizes on Matrigel. No spheroids could be formed for PDX line PH592 on Matrigel. Scale bar = 250 µm. **b** Viability for cultures from all PDX lines at day 14 comparing microfluidic (gray bar) and Matrigel (black bar) culture conditions. Microfluidic cultures showed significantly better viability (*p* < 0.05, *n* = 6) for all PDX lines except for PH704

Cultivation of cancer cells on Matrigel is a widely used strategy for forming and expanding spheroids; we therefore chose Matrigel-based cultures for benchmarking microfluidic cultures. We should note that the same media, organoid media, containing DMEM and FGF2, was used for both culture types and that the media contained 2% Matrigel. Figure 3a shows representative images of cells from 3 PDX lines after 7 days of culture on Matrigel-coated 6-well plates and in microfluidic devices. These images serve to contrast the heterogeneity of cancer cell aggregates on Matrigel to the relative uniformity of spheroids in microfluidic devices. In the case of a PDX model (PH592) of a more aggressive OC containing mesenchymal and epithelial cells, spheroid formation did not occur on Matrigel, but it was observed in microfluidic devices after 7 days of culture (middle panel, Fig. 3a). In fact, spheroids were successfully formed in microfluidic devices for all 6 PDX mouse models tested in this study (see Fig. S1 for images of additional PDX models on Matrigel and in microfluidic devices). Live/dead analysis, shown in Fig. 3b, revealed that viability in microfluidic cultures was significantly better than on Matrigel (*p* < 0.05) for five out of six PDX lines characterized in our study.

Parameters of spheroid viability and growth under three different culture conditions are summarized in Table 1. These data highlight the fact that microfluidic cultures were found to be superior for the majority of OC PDX models tested in this study.

**Table 1:** Comparison of viability and spheroid formation for the three culture conditions tested in this paper

PDX models	Histotype	Viability (%) at day 14			Spheroids Size (fold increase) at day 14	
		Matrigel	Standard	Microfluidic	Standard	Microfluidic
PH039	Serous	65 ± 16.5^a^	66 ± 8.3^a^	87 ± 8.0	1.3 ± 0.14^a^	1.7 ± 0.24
PH063	Serous	86 ± 4.8^a^	61 ± 3.6^a^	95 ± 2.4	1.1 ± 0.08^a^	1.6 ± 0.18
PH592	MMMT	83 ± 5.0^a^	60 ± 11.9^a^	88 ± 3.7	1.1 ± 0.11^a^	1.8 ± 0.16
PH704	Serous	82 ± 6.6 ^N.S.^	58 ± 5.1^a^	86 ± 2.7	1.1 ± 0.12^a^	1.3 ± 0.13
PH757	Serous	84 ± 1.3^a^	66 ± 15.2^a^	99 ± 0.3	1.7 ± 0.42^a^	2.5 ± 0.19
PH938	Serous	85 ± 8.7^a^	79 ± 12.9^a^	96 ± 1.6	1.3 ± 0.11^a^	1.6 ± 0.14

^a^Significantly lower (*p* < 0.05) compared to the values from the microfluidic culture format (*n* = 6)

*NS* Not significant difference (*p* < 0.05) compared to the values from the microfluidic device (*n* = 6)

### Assessing the phenotype of cancer spheroids

**Fig. 4:**
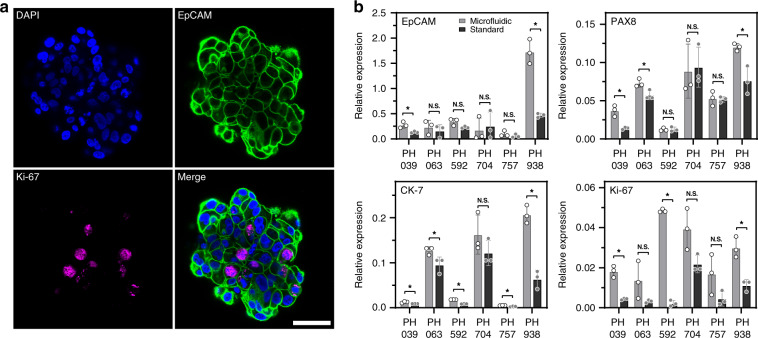
Phenotype assessment of OC spheroids. **a** A representative spheroid from a microfluidic device stained for an epithelial marker EpCAM and a proliferation marker Ki-67 as well as the nuclear dye DAPI. **b** PCR-RT results for the proliferation marker Ki-67 and epithelial markers PAX8, CK-7, and EpCAM. The results show similar or higher expression levels in microfluidic cultures compared to the standard culture method (*p* < 0.05, *n* = 3)

Moving beyond viability and spheroid growth, we wanted to assess the phenotype of OC cells by immunofluorescent staining for the epithelial marker EpCAM and proliferation marker Ki-67 in microfluidic devices. Strong expression of EpCAM confirmed that the cancer cells maintained an epithelial phenotype after 14 days of culture (Fig. 4a). While less prevalent, Ki-67 expression was observed within spheroids, thus corroborating the brightfield microscopy evidence of spheroid growth that was provided in Fig. 2.

In addition to immunostaining, we carried out RT-PCR analysis of the proliferation marker Ki-67 as well as epithelial and OC-specific genes (CK7, PAX8, and EpCAM) in microfluidic and standard (96-well plate with microwell insert) cultures. These results demonstrate that microfluidic cultures elicited higher EpCAM gene expression for two out of six PDX model, PAX8 gene expression for three out of six PDX models tested, and CK-7 expression was higher in microfluidic cultures for five out of six PDX models tested (Fig. 4b). The expression of the proliferation marker Ki-67 was higher in microfluidic cultures for three out of six PDX lines. Expression of target genes was normalized to the levels of the housekeeping gene GAPDH. We did not identify PDX lines that had significantly higher expression of phenotype markers in standard cultures compared to microfluidic cultures.

We also analyzed Ki67, EpCAM, CK7, Mucin-1, and PAX8 gene expression in three PDX lines cultured on Matrigel (Fig. S2). For 2 PDX lines (PH592 and PH938), Ki-67 expression was significantly higher in microfluidic cultures than it was in Matrigel. CK-7 gene expression was significantly higher in microfluidic cultures of PH938. In all other cases, we observed no significant differences in OC spheroid gene expression on Matrigel and microfluidic devices. These observations are important, given that Matrigel cultures are considered the gold standard for the formation, maintenance and expansion of cancer spheroids.

The results presented in this paper underscore the fact that OC spheroids form, grow and maintain phenotype in microfluidic cultures. The experiments that follow describe how microfluidic miniaturization and automation may be combined with cancer cultures to enable chemotherapy testing.

### Implementing a multichamber device to form spheroids from a minimal number of cells

**Fig. 5:**
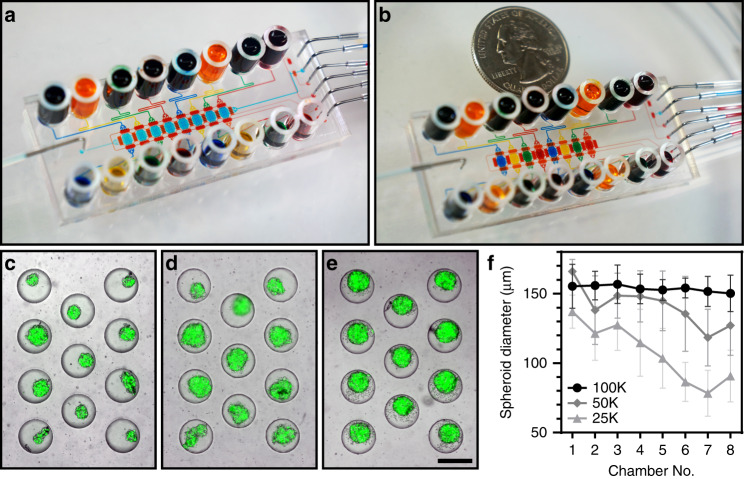
A multichamber microfluidic device. **a**, **b** Pictures showing a microfluidic device in serial perfusion (**a**) and parallel testing (**b**) modes. In serial perfusion, the device is configured to connect culture chambers serially. All culture chambers in (**a**) are filled with blue dye. In parallel perfusion, the device is reconfigured to sequester individual culture compartments and infuse them with dyes of different colors (blue, yellow, green, and red). **c**–**e** Images showing spheroids formed after seeding 25,000 (**c**), 50,000 (**d**), and 100,000 (**e**) cells into the device. Scale bar: 300 µm. **f** A graph showing spheroid diameter as a function of inoculation concentration for the 8 chambers of the device. Chamber 1 is closest to the inlet used for cell seeding, and chamber 8 is furthest. Spheroids were measured 24 h after seeding (*n* = 8)

As noted earlier, a key advantage of microfluidic cultures lies in minimizing the number of cells required to form spheroids and carry out drug testing. Our initial experiments were focused on optimizing device geometry, tissue digestion, cell seeding and culture protocols in simple, single chamber microfluidic devices. Subsequently, we designed and fabricated a multichamber microfluidic device consisting of 8 cell culture chambers, each containing an array of 11 microwells for spheroid formation (Fig. 5). Microfluidic valves (filled with red food dye in Fig. 5 for illustration purposes) allowed us to perfuse cell culture chambers in a serial or parallel fashion. Serial perfusion was useful for seeding all chambers from a suspension containing a small number of cells. Once cells were seeded, microvalves were actuated to sequester individual cell culture chambers, making them independent for drug testing experiments (see Fig. 5a, b for demonstrations of serial and parallel perfusion). Fig. S3 and Movie S1 show multichamber microfluidic device operation.

To highlight the utility of this microfluidic platform, different cell concentrations were titrated across device chambers, and spheroid formation was characterized. As shown in Fig. 5c–e, the spheroid size was dependent on the initial concentration of cells infused into the microfluidic platform. It was possible to form spheroids using as few as 25,000 cancer cells (MCF7 cells used in these experiments); however, spheroid diameter varied from ~140 µm in the culture compartment that was close to the sources of cells to ~70 µm in diameter in culture chamber 8, which was the furthest from the source of cells. Figure 5f demonstrates that infusion of 100,000 cells resulted in consistent seeding and spheroid formation in all 8 chambers of the microfluidic devices, irrespective of the proximity to the source of cells. In this case, spheroids of ~160-µm diameter were formed consistently throughout the chamber.

**Fig. 6:**
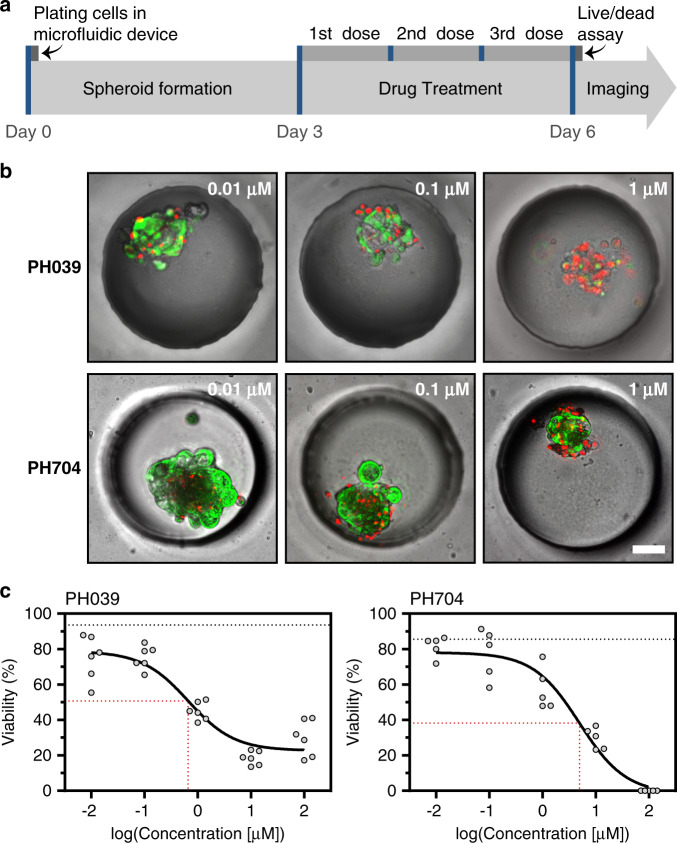
Drug treatment of cancer cells. **a** Drug treatment protocol for doxorubicin assays. **b** Micrographs showing a single spheroid at three different drug concentrations for two PDX lines. PH039 showed sensitivity to drug treatment at 0.659 µM, while PH704 sensitivity was lower at the same concentration. Scale bar = 50 µm. **c** Doxorubicin IC50 curves for sensitive (PH039, IC50–0.659 µM) and resistant (PH704, IC50–4.941 µM) ovarian cancer PDX lines. Red dotted lines denote IC50 values. Black dotted lines represent the viability of the negative control at day 6 (*n* ≥ 5)

In another set of experiments, it was determined that the formation and maintenance of spheroids in multichamber and single-chamber devices were similar (data not shown), and responses to doxorubicin in the PH039 line cultured in single and multichamber microfluidic devices were similar (IC50 = 0.659 µM for single and 0.832 µM for multichamber µM, see Fig. S4). With these data in hand, we deployed a multichamber microfluidic device to characterize responses to a common chemotherapeutic, doxorubicin. When testing the cytotoxicity of chemotherapy, live/dead staining was utilized—this is the same approach as described earlier in this paper in the context of viability analysis. Fig. 6 shows representative images of cancer spheroid viability following exposure to different concentrations of doxorubicin. To account for the 3D distribution of live and dead cells in the volume of a spheroid, we collected images at different focal planes and then created a compressed projection of these images on a 2D plane. These projections were analyzed using ImageJ to quantify green (live) and red (dead) fluorescent cells. Fig. 6a describes the time course of drug treatment and presents responses from 2 PDX models to treatment with doxorubicin. Drug treatment commenced at day 3 of culture, which was after spheroids had formed, and then the treatment proceeded for 3 days, with daily media exchange and daily addition of fresh doxorubicin. Line PH704 had an IC50 of ~5 µM, so it was considered less responsive, whereas PH039, with an IC50 of 0.659 µM, was more sensitive (Fig. 6b, c). Importantly, IC50 curves were established using one device based on the input of a small number of OC cells.

#### Is the formation of spheroids important for chemotherapy testing in vitro?

Given chemotherapy may have a cytotoxic effect where cells within spheroids undergo apoptosis/necrosis, as shown in Fig. 6b. However, other drugs may elicit cytostatic effects where minimal cell death is observed but spheroid growth is inhibited. Figs. S5 and S6 compare the drug responses of PDX model PH757 and all PDX lines to doxorubicin and gemcitabine, respectively. As highlighted in these images, doxorubicin had a cytotoxic effect, with more than 50% of cells were dead after exposure to a 0.65 µM concentration of the drug. In contrast, gemcitabine concentrations as high as 100 µM had only a minor cytotoxic effect; instead, treatment with this drug abrogated spheroid growth (Fig. S5). These results highlight that spheroid growth or shrinkage may provide important information pertaining to mechanisms of chemotherapeutic action. Such information is difficult to glean from Matrigel-based cultures or other cancer culture systems where spheroids are not uniform in size and are not arranged in an array format.

## Conclusions

This paper demonstrates the cultivation of OC spheroids in microfluidic devices. Comparison of three different culture conditions (microfluidic, standard 3D and Matrigel) revealed that microfluidic cultures were superior to standard (96-well plate) 3D cultures in terms of viability, proliferation and phenotypic gene expression of OC spheroids. In addition, the microfluidic platform enabled the formation of uniform OC spheroids from all 6 PDX models tested, whereas Matrigel-based cultures elicited a wide distribution of spheroid sizes and, in the case of two of the PDX models, resulted in no spheroid formation at all. Thus, one finding of our paper is that PDMS-based microfluidic devices represent an excellent platform for the formation and maintenance of primary OC spheroids.

Beyond spheroid formation and phenotype maintenance, we demonstrate that the use of on-board microvalves enables spheroid formation in an 8-chamber microfluidic device based on an input of as few as 100,000 cells. This number of cells is insufficient for testing multiple experimental conditions using standard culture approaches (e.g., 96-well plate format). To demonstrate the utility of a multichamber microfluidic platform, we tested different concentrations and established IC50 values for doxorubicin within the same device based on infusion of ~100,000 cells. Given that ~100,000 is at the lower limit of the number of cells present in a fine-needle aspirate, we are well positioned for future studies aimed at testing drug responses based on biopsies from cancer patients. Our vision for the future is to use the microfluidic cancer spheroid culture platform to test chemotherapy responses and inform oncologists’ selection of a therapeutic regimen for individual cancer patients.

## Materials and methods

### PDX lines used in the study

Patient-specific tumorigenesis was established in severe combined immunodeficient (SCID) mice as described by us previously^6^.

### Organoid media

We used a custom formulation of media to maintain cells in all three culture methods: Matrigel, Standard and Microfluidic, and we named it “Organoid media”. The media was composed of DMEM with high glucose and pyruvate (Gibco), 1% B-27 supplement (Gibco), 50 μg/mL ascorbic acid (Sigma Aldrich), 20 μg/mL insulin (Sigma Aldrich), 0.25 μg/mL hydrocortisone, 100 ng/mL FGF2 growth factor (R&D Systems), 100 nM *all-trans-*retinoic acid (Sigma Aldrich), 10 μM ROCK inhibitor (Tocris), 2% Matrigel growth factor-reduced (B&D Systems), and 1% antibiotic-antimycotic (100×, Gibco).

### Tumor tissue digestion

The workflow for tumor digestion and cell preparation is shown in Fig. S7. Following excision of each tumor from a mouse, we obtained a piece of tumor >2 cm in diameter. The tissue was first cleaned by submersion in 1X Hank’s balanced salt solution without Ca^2+^ and Mg^+^ (HBSS, Sigma Aldrich) to remove the blood and outer mouse stroma. Then, tissue was submerged in 5 mL of DMEM/F12 (Gibco) on a culture plate and chopped into small fragments using a scalpel. Tissue fragments were enzymatically digested by incubation on a rotary shaker at 37 °C and 85 rpm for 60 min in a solution containing 8 mL of DMEM/F12 (Gibco), 2 mL of bovine serum albumin (BSA, Sigma Aldrich), 200 μL of a mixture of collagenase (3000 U/mL, Sigma Aldrich) and hyaluronidase (1000 U/mL, Sigma Aldrich) and 100 μL of penicillin-streptomycin (100×, Gibco). Digested cells were passed through a 40-μm cell strainer (Fisher Scientific) to remove large tissue chunks. Small cell aggregates (<100 µm) were kept in fresh organoid media.

### Formation of spheroids on Matrigel

Throughout this study, we used Matrigel cultures for two purposes: (1) to form spheroids prior to seeding into microfluidic devices and (2) as a gold standard spheroid culture for benchmarking microfluidic spheroid cultures. For both purposes, 6-well plates were coated with 300 μL of Matrigel (BD Biosciences) and incubated at 37 °C for at least 30 min before cell seeding to induce gelation. Cell aggregates obtained from tumor digestion were seeded onto Matrigel at a concentration of 1 × 10^6^ cells per well and then were maintained for at least three days to induce aggregation and spheroid formation. Cells were maintained in an incubator at 37 °C and 5% CO_2_ in organoid media. The presence of growth factor FGF2 in the media has been shown previously to induce spheroid formation^38^. For the purposes of seeding into microfluidic devices and the standard method, cell aggregates were collected from Matrigel-coated plates as described in the next section after 3 days of culture. Alternatively, Matrigel-based cultures were maintained for 14 days and were used for benchmarking of our microfluidic cancer cultures.

### Formation of spheroids in large volume microwell cultures

In addition to spheroid cultures on Matrigel, we also employed 3D cultures on 96-well plates (termed standard cultures in this paper) in a larger volume (180 µL) for comparison to microfluidic 3D cultures, which have a chamber volume of 6.3 µL. We employed polydimethylsiloxane (PDMS) inserts that were made to fit into wells of a 96 well plate (5 mm diameter and 1 mm height). These inserts were molded using soft lithography approaches to contain 80 cylindrical microwells; each microwell was 250 µm in diameter and 300 µm in depth. Wells of a 96-well plate containing inserts were made to prevent adhesion of cells by treatment with Pluronic F-127 at 1% in 1X PBS overnight.

For cell seeding in standard cultures, spheroids from Matrigel were dissociated into single cells. Spheroids were collected from Matrigel-coated 6-well plates by placing 1 mL of dispase (Stem Cell Technology) and 100 μL of DNase I (Sigma Aldrich) into each well and incubating for 2 h. Afterwards, 1 mL of cold 1× HBSS with 2% fetal bovine serum (FBS, Gibco) was added into each well to stop the effects of displace, and the resultant solution was transferred into a conical 15 mL tube, where it was combined with 2 mL of 1× HBSS. Cells were then centrifuged at 1200 rpm for 5 min at 4 °C, after which the supernatant was removed, and the cell pellet was resuspended in 1 mL of organoid media. The protocol described above resulted in dissociation of OC spheroids into a single cell suspension or into clusters of a few (2–4) cells.

Cancer cells were suspended in organoid media at 1 × 10^5^ cells per 200 μL of media and then were placed into wells of a 96-well plate containing PDMS inserts as standard culture. After 10 min, media containing cells outside of microwells was removed, and 200 μL of fresh media was added. After this, the media was exchanged every 24 h.

### Formation of cultures in microfluidic devices

For microfluidic cultures, single OC cells were prepared in the same fashion as for standard cultures. Pluronic F-127 solution was injected into the microfluidic device, placed in a vacuum desiccator to remove air trapped inside the device and placed at 4 °C overnight before cell seeding. A total of 4 × 10^5^ cells were deposited in the inlet of the device and allowed to flow through the culture chamber until the bottom of the microwells were filled with cells. After filling microwells, excess cells were removed by washing the device with fresh organoid media. Cells were cultured in microfluidic and standard conditions at 37 °C for 14 days for spheroid growth, viability, PCR and immunofluorescence, and three days for cytotoxicity experiments, changing media every 24 h.

### Fabrication of a single chamber microfluidic device

Microfluidic devices were designed using CAD software (AutoCAD 2018, Autodesk Inc.) and were fabricated in PDMS using well-established photolithography (Fig. S8) and soft lithography methods (Fig. S9)^39^. The single-chamber device consisted of two PDMS layers, the top layer containing flow channels and a cell culture chamber and a bottom layer containing an array of 19 microwells. Each cylindrical microwell had the following dimensions: 250 µm in diameter and 300 µm in depth. A typical single chamber microfluidic device is shown in Fig. 1. It contained seeding/transport channels that were 300 µm in height and 500 µm in width, which were connected to a rectangular cell culture chamber with dimensions of 3 × 7 mm. The top and bottom PDMS layers were bonded by 2 min of exposure to oxygen plasma (PDC-001, Harrick Plasma).

### Fabrication of a multichamber microfluidic device with integrated microvalves

Once successful maintenance of cancer spheroids in a single chamber microfluidic device was demonstrated, we proceeded to fabricate a multichamber microfluidic device. This device was designed to enable spheroid formation from a minimal amount of cancer tissue, similar to what is present in a core from an 18-gauge needle commonly used for biopsy collection. Each device contained 8-culture chambers, which themselves each contained 11 microwells as well as microvalves for orthogonal control of flow, which enabled the following strategies: (1) serial flow for seeding cells and (2) parallel flow for sequestering individual chambers and testing drugs. These multichamber devices were comprised of three PDMS layers: (1) a bottom layer containing cylindrical microwells that were 250 µm in diameter and 300 µm deep, (2) a flow layer containing transport channels and culture chambers and (3) a control layer containing microvalves.

Each PDMS layer was molded using its own master wafer (see Fig. S8). To make layers 1 and 3, Si wafers were coated with negative photoresist, SU-8 2100 (MicroChem) and SU-8 2050 (MicroChem), to achieve thicknesses of 300 and 50 µm. A soft bake was performed for 10 min at 65 °C and 90 min at 95 °C on a hot plate. Afterwards, both wafers were exposed to UV light through a photomask with the desired structures using a mask aligner (UV-KUB 3, Kloé). A postexposure bake was performed for 5 min at 65 °C and 15 min at 95 °C. Development was performed using direct submersion on SU-8 developer (MicroChem) until unexposed photoresist was completely removed.

Flow mold was fabricated using positive (AZ50XT, MicroChemicals) and negative photoresists (SU-8 2050). First, a plasma-treated Si wafer was coated with a 60 µm layer of positive photoresist; then, it was soft baked and exposed using the mask aligner. The master wafer was developed and then placed on a hotplate at 130 °C for 30 min before being baked overnight in a convection oven at 210 °C. Heat treatment was necessary to reflow positive photoresist from rectangular to rounded features needed for effective closing of the flow channels. The master wafer was then plasma treated for 1.5 min and patterned with a 100 µm layer of negative resist. The master wafer fabrication process was similar for the bottom layer that contained microwells and the control layer that was used for pneumatic actuation of the flow layer. To improve the adhesion of structures to the substrate, a hard bake was performed on a hot plate at 160 °C for 30 min for all molds. Finally, all molds were exposed to chlorotrimethylsilane (Sigma Aldrich) for 30 min inside a vacuum desiccator and stored on petri dishes until further use. The fabrication of molds is summarized in Fig. S8.

Once master wafers for each of the three layers were fabricated, steps were taken to replicate each mold in PDMS and then three PDMS layers were assembled into a functional device. The fabrication of the microwell, flow and control PDMS layer and assembly of these layers into a microfluidic device are summarized in Fig. S9. First, 12.6 g of PDMS (Sylgard 184 silicone elastomer kit, Dow Corning) was prepared at a 20 to 1 ratio of base to curing agent, and then it was poured on the flow mold to achieve a thickness of ~150 µm. When making the control layer, 24 g of PDMS prepolymer at a 5 to 1 base to curing agent ratio was poured on top of the control master wafer to achieve a height of ~4 mm. The bottom PDMS later, containing arrays of microwells, was fabricated by dispensing 14.3 g of PDMS prepolymer (10:1 ratio) to create a thickness of ~2 mm in the resultant cured PDMS layer. Master wafers for the control and microwells layers were degassed in a vacuum desiccator for 10 min. All molds were baked for 23 min in a convection oven at 80 °C for partial curing. After partial curing, PDMS from the control and the microwells molds were peeled off, devices were cut out, and holes in inlets/outlets were punched. The PDMS layer containing microwells was set aside. Control PDMS replicas were aligned on top of the flow mold under a stereoscope (Zoom 2000, Leica Microsystems) and baked together for 90 min. Subsequently, devices were peeled off from the flow mold, and inlet/outlet holes were punched. The flow/control device was placed on a clean silicon wafer with the channels facing up, and microwells PDMS replica were aligned on top of it. The three-layer PDMS device was baked for at least 16 h. Finally, 6-mm diameter glass cylinders were bonded on top of each chamber inlet/outlet using uncured PDMS and baking for 20 min at 80 °C. For single chamber devices, 10 mm glass cylinders were used.

### Operating multichamber microfluidic devices

Before experiments, all PDMS microfluidic devices were treated with Pluronic F-127. First, glass cylinders are filled with Pluronic F-127 at 1% in 1X PBS. For degassing, all inlets and outlets were blocked, and a tube filled with Pluronic F-127 was connected to a single inlet. The solution was pressurized at 3 psi until all air was removed from the structures. By applying pressure and blocking inlets and outlets, air diffused through gas permeable PDMS, resulting in all structures being filled with Pluronic F-127 in the absence of air bubbles. Afterwards, chambers and channels were first washed using 1X PBS, which was followed by a second washing step using organoid media. Cells were seeded afterwards.

For the valve-enabled device, all control microchannels were filled with DI water and pressurized to 8 psi to remove air trapped inside the structures. Control lines were connected to a set of solenoid valves to control activation using a custom-made graphic user interface (GUI) made in LabVIEW (v2017, National Instruments). For valve activation, 30 psi of pressurized air was applied to control lines to pinch off flow channels.

### Spheroid growth rate calculation

For all culture conditions, spheroids were tracked for 14 days, and bright-field images were acquired at days 1, 3, 7, and 14 after seeding. All spheroids in all images were traced using ImageJ to estimate the area at each time point. The area at every time point was normalized to the area at day 1 to track spheroid growth and to compare it between culture conditions. At day 14 after seeding, a live/dead assay was performed for all conditions.

### Testing viability of cancer cultures

For viability assays, microfluidic devices, Matrigel plates and standard culture plates were washed twice with 1X PBS to remove all media. The staining solution was prepared in a 15 mL tube by mixing 10 mL of 1X PBS, 20 µL of ethidium homodimer and 5 µL of Calcein-AM (Live/Dead Viability/Cytotoxicity Kit, Thermo Fisher Scientific). Then, 200 µL of staining solution was added to all culture conditions and incubated for 30 min at room temperature. The staining solution was washed using 1X PBS. A Z-stack was acquired for all conditions in brightfield and fluorescence channels for calcein (ex/em 494/517 nm) and ethidium homodimer (ex/em 528/617 nm) using an inverted microscope (IX-83, Olympus), and images were captured in 11 focal planes separated by 10 µm for a total range of 100 µm. A deconvolution function was used to create a single image with a projection of fluorescence signals from all images in the Z-stack while eliminating the blur from out-of-focus signals. Projection images were analyzed using ImageJ by comparing the area of the calcein signal with the area of the ethidium homodimer signal for each spheroid to determine viability under each condition.

### Immunofluorescence and PCR analysis of cancer spheroid cultures

After cultivation in microfluidic devices for 7 and 14 days, spheroids were washed with 1X PBS and fixed with 4% paraformaldehyde (Electron Microscopy Sciences) for 1 h at room temperature. Afterwards, devices were washed twice with 1X PBS. Five hundred microliters of a blocking solution containing Triton X at 0.5% (Invitrogen) and BSA (Sigma Aldrich) at 3% was injected into the devices and incubated overnight. Then, primary antibodies against Ki-67, diluted 1–1000 in blocking buffer, EpCAM, diluted 1–50, and pan-cytokeratin, diluted 1–50, were added to the microfluidic devices and incubated overnight. Secondary antibodies were injected and incubated for 3 h. Excess secondary antibody solution was removed by flushing with fresh 1X PBS 5 times. Then, mounting media with DAPI (Abcam) was infused into the channel for 5 min. Afterwards, micrographs were acquired using a confocal microscope (LSM 780, Zeiss Microscopy) with a ×20 objective.

For PCR experiments, cells were harvested on day 14. Gene expression in PDX spheroids was analyzed using real-time RT-PCR. Total RNA was purified using a High Pure RNA Isolation Kit (Roche Diagnostics) according to the manufacturer’s instructions. Afterwards, random hexamer primed cDNA synthesis was generated from purified RNA using a Revert Aid first-strand cDNA synthesis kit (Roche Diagnostics). Quantitative real-time PCR was performed with the cDNA for 40 cycles on a PCR system (QuantStudio 3 and 5 Real-Time PCR Systems, Applied Biosystems). To quantify cDNA amplification, FastStart Universal SYBR Green Master (ROX) (Roche Diagnostic) melting curve analysis was used to confirm PCR specificity. Each reaction was performed three times by mixing spheroids from two microfluidic devices. All primers were acquired from Integrated DNA Technologies and have the following sequences: human CK-7 forward, 5′-caccatgtccatccacttca-3′, reverse, 5′-gaggccgtagaggctgct-3′; human PAX8 forward, 5′-caggctgagatcgacaacatc-3′, reverse, 5′-cttggcacgagcatcctt-3′; human EpCAM forward, 5′-aatcgtcaatgccagtgtactt-3′, reverse, 5′-tctcatcgcagtcaggatcataa-3′; human GAPDH forward, 5′-ggtggtctcctctgacttcaaca-3′, reverse, 5′-gtggtcgttgagggcaatg-3′; and human Ki-67 forward, 5′-gacagaggttcctaagagag-3′, reverse, 5′-aacaatcagatttgcttccg-3′. Human Mucin-1 primers, forward and reverse, were acquired from Qiagen (cat. no. QT00015379). Relative expression was quantified using the ∆Ct method. Target gene (Ki-67, CK7, PAX8, and EpCAM) expression was normalized to that of the housekeeping gene GAPDH.

### Drug exposure and cytotoxicity testing

Spheroids were exposed to standard chemotherapy agents, doxorubicin and gemcitabine, in single- and multichamber microfluidic devices. Spheroids residing in different chambers of a multichamber device or in different single-chamber devices were exposed to drug concentrations ranging from 0.01 to 100 µM. Medium with the drug was changed daily with the entire length of the exposure lasting 3 days. Live/dead staining, discussed above, was used to quantify cytotoxicity and establish the half-maximal inhibitory concentration (IC50) for both gemcitabine and doxorubicin.

### Statistical analysis

Student’s *t*-tests were used for statistical analysis of all experiments. A minimum of three biological replicates were used for each condition, and the standard deviation is presented as error bars. The number of biological duplicates and significance (*p*) value threshold used for each experiment are listed in figure captions.

## Supplementary information

Movie S1: Multiplex device testing with food dyes

Supplemental info

## References

[1] Siegel RL, Miller KD, Jemal A (2018). Cancer statistics, 2018. CA. Cancer J. Clin..

[2] Bowtell DDL (2010). The genesis and evolution of high-grade serous ovarian cancer. Nat. Rev. Cancer.

[3] Ozols RF (2003). Phase III Trial of Carboplatin and Paclitaxel Compared With Cisplatin and Paclitaxel in Patients With Optimally Resected Stage III Ovarian Cancer: A Gynecologic Oncology Group Study. J. Clin. Oncol..

[4] AlHilli MM (2016). In vivo anti-tumor activity of the PARP inhibitor niraparib in homologous recombination deficient and proficient ovarian carcinoma. Gynecol. Oncol..

[5] Glaser G (2015). Conventional chemotherapy and oncogenic pathway targeting in ovarian carcinosarcoma using a patient-derived tumorgraft. PLoS ONE.

[6] Weroha SJ (2014). Tumorgrafts as in vivo surrogates for women with ovarian cancer. Clin. Cancer Res..

[7] Mehta G, Hsiao AY, Ingram M, Luker GD, Takayama S (2012). Opportunities and challenges for use of tumor spheroids as models to test drug delivery and efficacy. J. Control. Release.

[8] Weiswald L-B, Bellet D, Dangles-Marie V (2015). Spherical cancer models in tumor biology. Neoplasia.

[9] Boj SF (2015). Organoid models of human and mouse ductal pancreatic. Cancer Cell.

[10] Huang L (2015). Ductal pancreatic cancer modeling and drug screening using human pluripotent stem cell– and patient-derived tumor organoids. Nat. Med..

[11] Nuciforo S (2018). Organoid models of human liver cancers derived from tumor needle biopsies. Cell Rep..

[12] Li X (2018). Organoid cultures recapitulate esophageal adenocarcinoma heterogeneity providing a model for clonality studies and precision therapeutics. Nat. Commun..

[13] Hill SJ (2018). Prediction of DNA repair inhibitor response in short-term patient-derived ovarian cancer organoids. Cancer Discov..

[14] Kopper O (2019). An organoid platform for ovarian cancer captures intra- and interpatient heterogeneity. Nat. Med..

[15] Nath S, Devi GR (2016). Three-dimensional culture systems in cancer research: focus on tumor spheroid model. Pharmacol. Ther..

[16] Cui X, Hartanto Y, Zhang H (2017). Advances in multicellular spheroids formation. J. R. Soc. Interface.

[17] Ryu N-E, Lee S-H, Park H (2019). Spheroid culture system methods and applications for mesenchymal stem cells. Cells.

[18] Moshksayan K (2018). Spheroids-on-a-chip: recent advances and design considerations in microfluidic platforms for spheroid formation and culture. Sens. Actuators B Chem..

[19] Ahn, J. et al. 3D microfluidic bone tumor microenvironment comprised of hydroxyapatite/fibrin composite. *Front. Bioeng. Biotechnol*. **7**, 168 (2019). https://www.frontiersin.org/articles/10.3389/fbioe.2019.00168/full. 10.3389/fbioe.2019.00168 PMC6653063 31380359

[20] Fan Q (2017). A novel 3-D bio-microfluidic system mimicking in vivo heterogeneous tumour microstructures reveals complex tumour–stroma interactions. Lab. Chip..

[21] Trujillo-de Santiago G (2019). The tumor-on-chip: recent advances in the development of microfluidic systems to recapitulate the physiology of solid tumors. Materials.

[22] Zhang W, Huang P (2011). Cancer-stromal interactions. Cancer Biol. Ther..

[23] Parlato S (2017). 3D Microfluidic model for evaluating immunotherapy efficacy by tracking dendritic cell behaviour toward tumor cells. Sci. Rep..

[24] Lee, S. W. L. et al. Characterizing the role of monocytes in T cell cancer immunotherapy using a 3D microfluidic model. *Front. Immunol*. **9**, 416 (2018). https://www.frontiersin.org/articles/10.3389/fimmu.2018.00416/full. 10.3389/fimmu.2018.00416 PMC5845585 29559973

[25] Park, D. et al. High-Throughput Microfluidic 3D Cytotoxicity Assay for Cancer Immunotherapy (CACI-IMPACT Platform). *Front. Immunol*. **10**, 1133 (2019). https://www.frontiersin.org/articles/10.3389/fimmu.2019.01133/full. 10.3389/fimmu.2019.01133 PMC6546835 31191524

[26] Adriani G (2016). Microfluidic models for adoptive cell-mediated cancer immunotherapies. Drug Discov. Today.

[27] Kim C (2011). Generation of core-shell microcapsules with three-dimensional focusing device for efficient formation of cell spheroid. Lab. Chip..

[28] Sabhachandani P (2016). Generation and functional assessment of 3D multicellular spheroids in droplet based microfluidics platform. Lab. Chip..

[29] Aijian AP, Garrell RL (2015). Digital microfluidics for automated hanging drop cell spheroid culture. J. Lab. Autom..

[30] Rismani Yazdi S (2015). Adding the ‘heart’ to hanging drop networks for microphysiological multi-tissue experiments. Lab. Chip..

[31] Wu LY, Di Carlo D, Lee LP (2008). Microfluidic self-assembly of tumor spheroids for anticancer drug discovery. Biomed. Microdevices.

[32] Fu C-Y (2014). A microfluidic chip with a U-shaped microstructure array for multicellular spheroid formation, culturing and analysis. Biofabrication.

[33] Chen Y, Gao D, Liu H, Lin S, Jiang Y (2015). Drug cytotoxicity and signaling pathway analysis with three-dimensional tumor spheroids in a microwell-based microfluidic chip for drug screening. Anal. Chim. Acta..

[34] Patra B (2013). A microfluidic device for uniform-sized cell spheroids formation, culture, harvesting and flow cytometry analysis. Biomicrofluidics.

[35] Kim C, Bang JH, Kim YE, Lee SH, Kang JY (2012). On-chip anticancer drug test of regular tumor spheroids formed in microwells by a distributive microchannel network. Lab. Chip..

[36] Rajshekar SK (2013). Malignant mixed Mullerian tumour of the uterus. Ecancermedicalscience.

[37] Haque A (2016). Cell biology is different in small volumes: endogenous signals shape phenotype of primary hepatocytes cultured in microfluidic channels. Sci. Rep..

[38] Baker LA, Tiriac H, Clevers H, Tuveson DA (2016). Modeling pancreatic cancer with organoids. Trends Cancer.

[39] Gonzalez-Suarez AM, Peña-del Castillo JG, Hernández-Cruz A, Garcia-Cordero JL (2018). Dynamic generation of concentration- and temporal-dependent chemical signals in an integrated microfluidic device for single-cell analysis. Anal. Chem..

