# Supplementary material for: A microfluidic platform for cultivating ovarian cancer spheroids and testing their responses to chemotherapies
Source: Microsyst Nanoeng. 2020 Oct 19;6:93. doi: 10.1038/s41378-020-00201-6 (PMC8433468; doi:10.1038/s41378-020-00201-6)
Supplement: Supplementary file 2 — Supplemental info [file 41378_2020_201_MOESM2_ESM.docx]

**Supplementary information for**

**A Microfluidic Platform for Cultivating Ovarian Cancer Organoids and Testing Chemotherapy Responses**

Neda Dadgar^1§^, Alan M. Gonzalez-Suarez^1§^, Pouria Fattahi^1^, Xiaonan Hou^2^, S. John Weroha^2^, Alexandre Gaspar-Maia^3^, Gulnaz Stybayeva^1*^ and Alexander Revzin^1*^

*^1^Department of Physiology and Biomedical Engineering, Mayo Clinic, Rochester, MN, USA 55905*

*^2^Department of Medical Oncology, Mayo Clinic, Rochester, MN, USA 55905*

*^3^Department of Laboratory Medicine and Pathology, Mayo Clinic, Rochester, MN, USA 55905*

^§^Equally contributing authors.

^*^Corresponding authors: [stybayeva.gulnaz@mayo.edu](mailto:stybayeva.gulnaz@mayo.edu), [revzin.alexander@mayo.edu](mailto:revzin.alexander@mayo.edu)


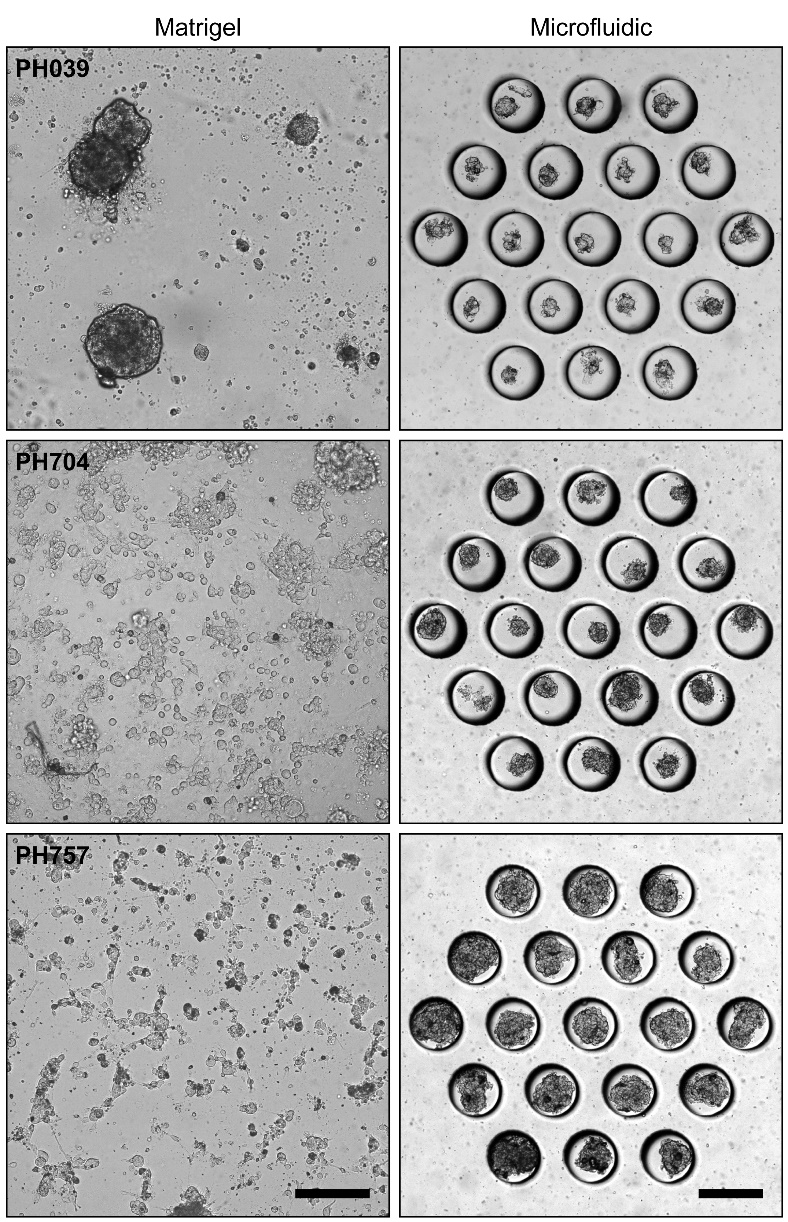


**Figure S1.** **Comparison of spheroid formation on Matrigel and in microfluidic devices.** These micrographs show spheroid formation for 3 PDX lines, PH039, 704 and 757 that are not described in Figure 2. Matrigel scale bars = 200 µm, microfluidic device scale bar = 300 µm.


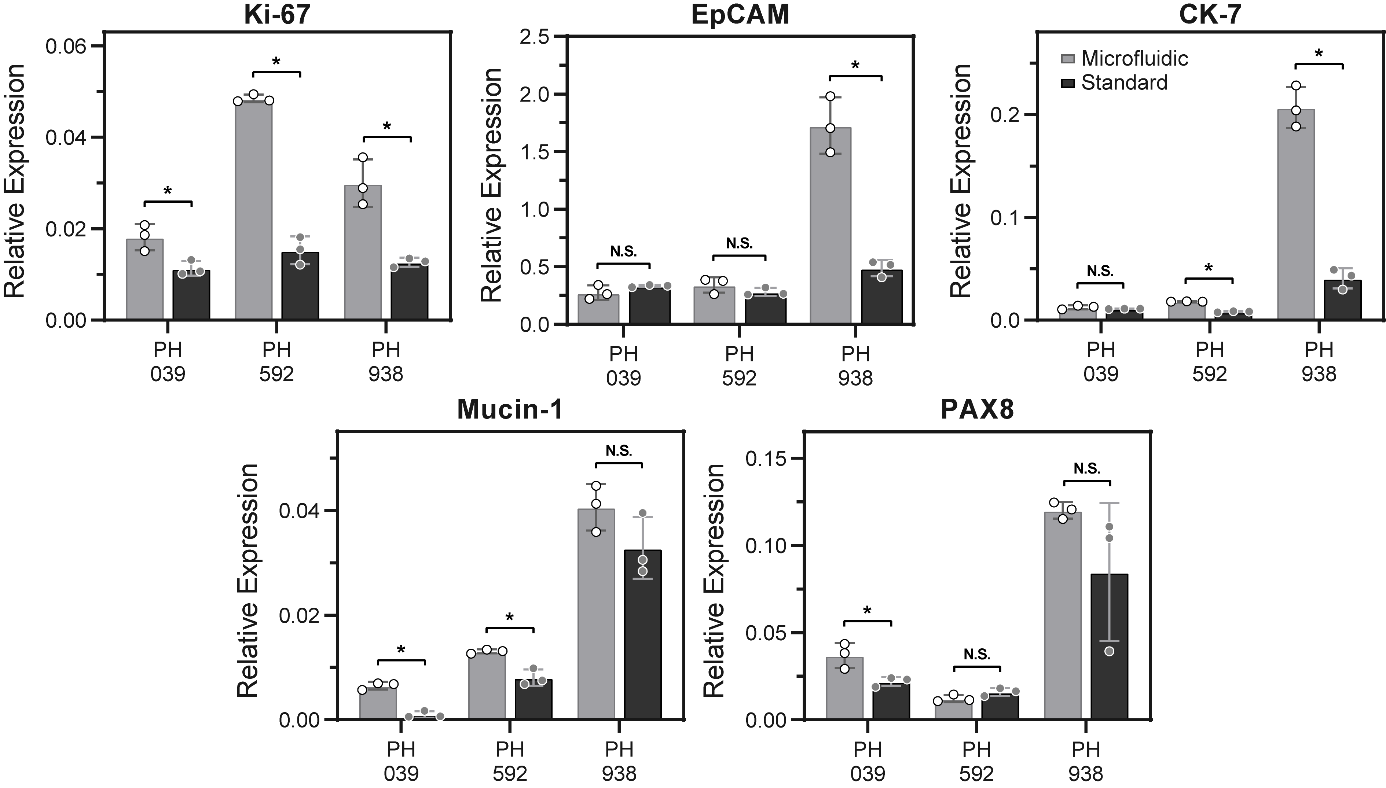


**Figure S2.** Comparison of gene expression for ovarian cancer organoids cultured on Matrigel and in microfluidic devices. Statistical significance p<0.05.


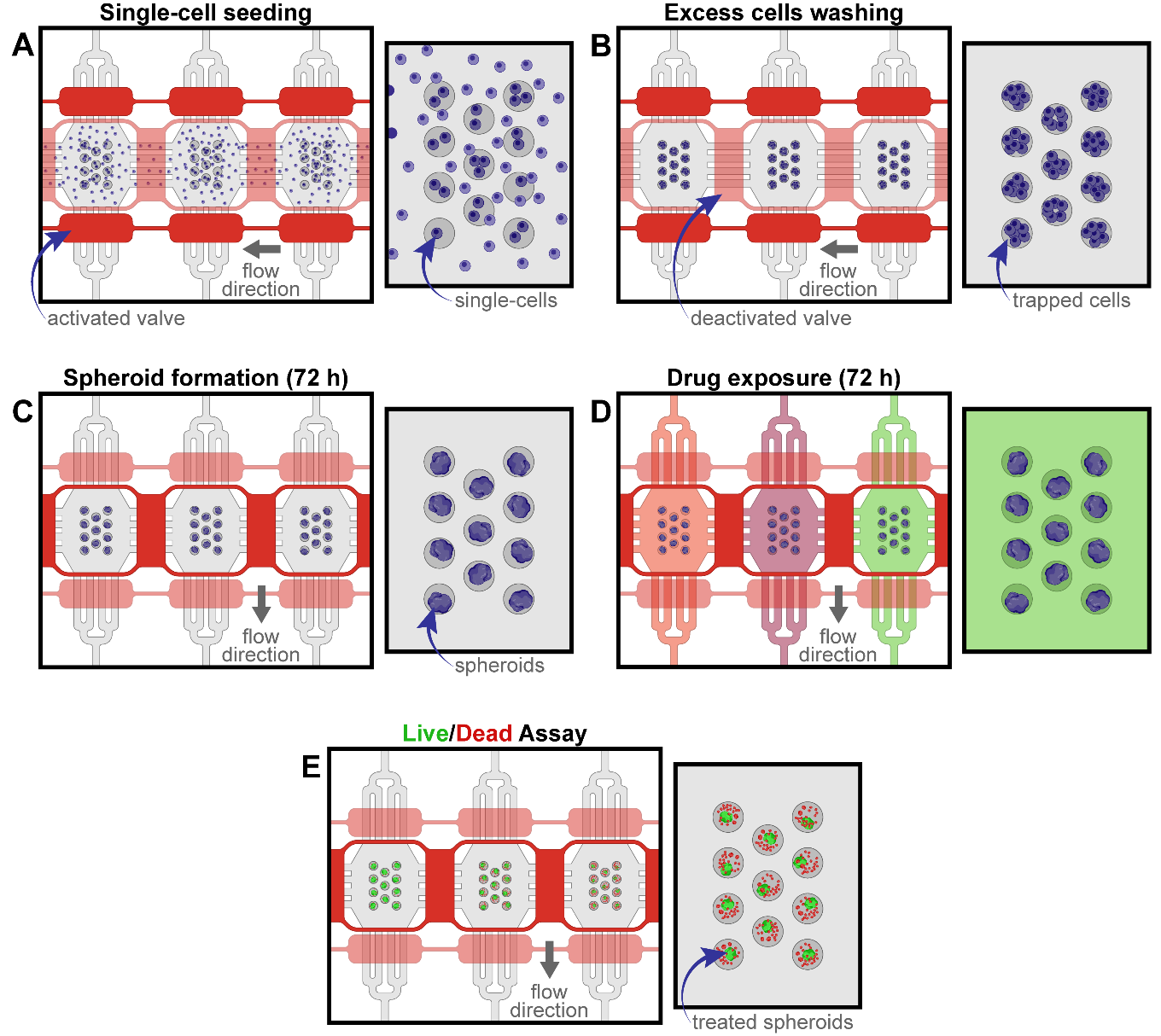


**Figure S3: Multi-chamber microfluidic device operation.** (**A**) After device preparation, single cells are seeded in a serial fashion by deactivating lateral valves and until all wells are evenly filled. (**B**) Excess cells are washed away in the same manner. (**C**) Lateral valves are activated, and top/bottom valves deactivated to allow fresh media to flow through chambers. Cells are cultured for 72 h to allow spheroid formation. (**D**) After this time, all chambers are stimulated with different concentrations of a specific drug for 72 h. (**E**) Finally, live/dead assay is performed to determine effect of drug on spheroids.


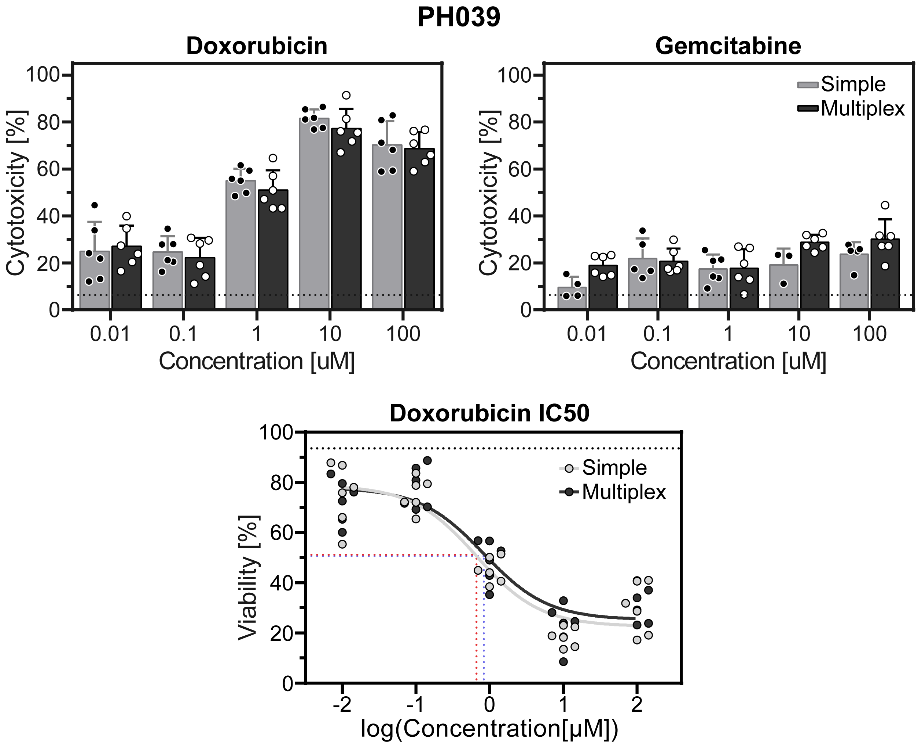


**Figure S4:** **Comparison of drug responses in single and multi-chamber culture devices.** Top graphs show drug cytotoxicity after exposure to different concentrations of doxorubicin or gemcitabine in single and multiplex microfluidic devices. For both devices results are similar, indicating no difference between cell culture conditions among them. Bottom graph shows similar IC50 calculations for both devices: 0.659 µM for simple (red dotted line) and 0.832 µM for multiplex device (blue dotted line). Black dotted lines represent viability of negative control at day 6.


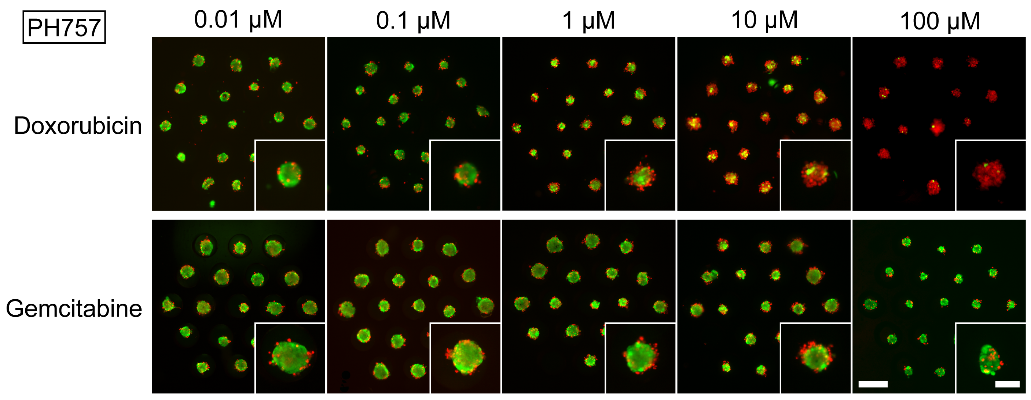


**Figure S5:** **Demonstration of different chemotherapy drugs having cytostatic and cytostatic effects on cancer spheroids.** Live/dead assay micrographs after drug treatments to a PDX line. Fluorescence micrographs show live (green) and dead (red) cells after drug exposure with different concentrations of doxorubicin and gemcitabine to PDX938 line. Fluorescence images scale bar = 300 µm, inserts scale bar = 100 µm.


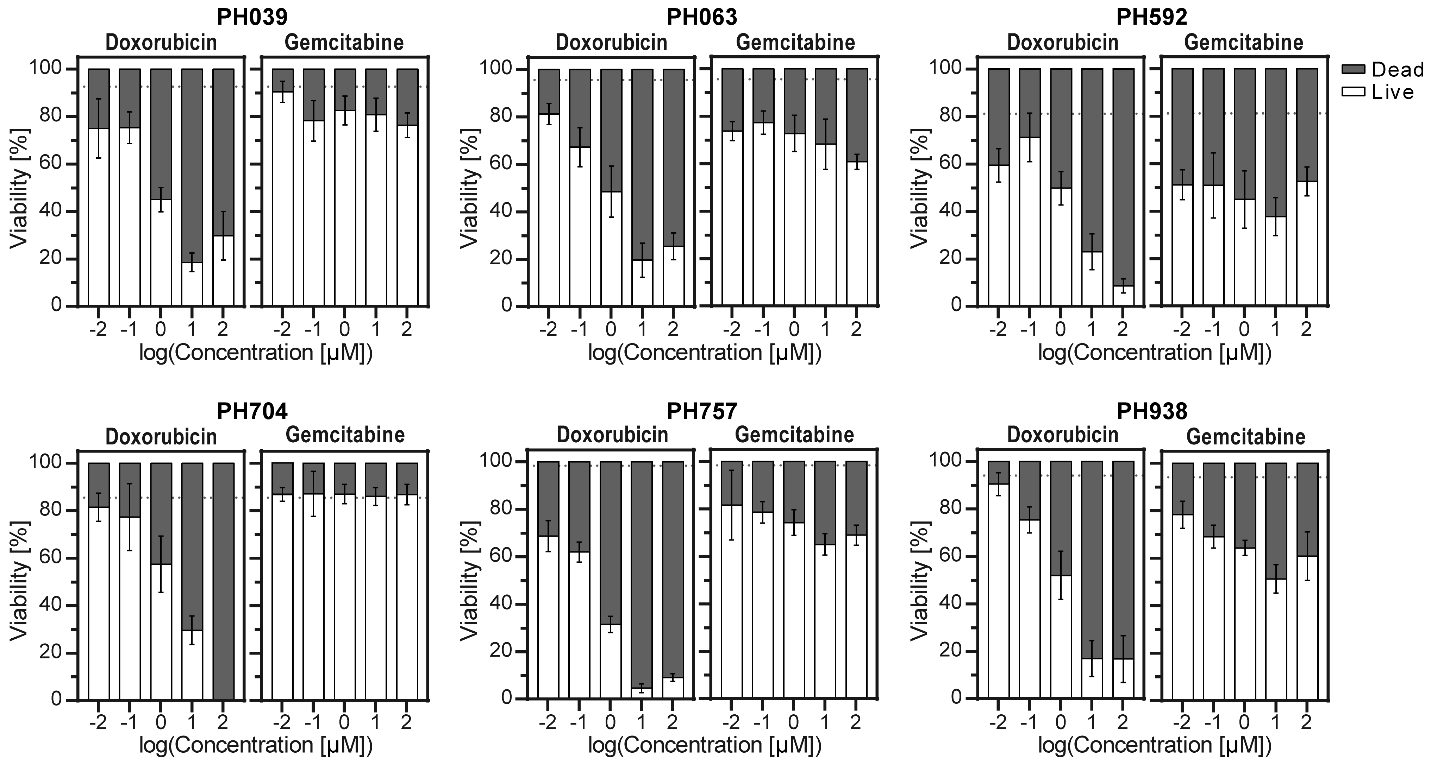


**Figure S6:** **Live/dead percentage of cells after drug treatment.** Graphs showing live/dead assay results after drug treatments to all PDX lines with different concentrations of doxorubicin and gemcitabine. Doxorubicin graphs show a cytotoxic effect at increasing drug concentrations, while gemcitabine effect is comparable throughout the different concentrations.


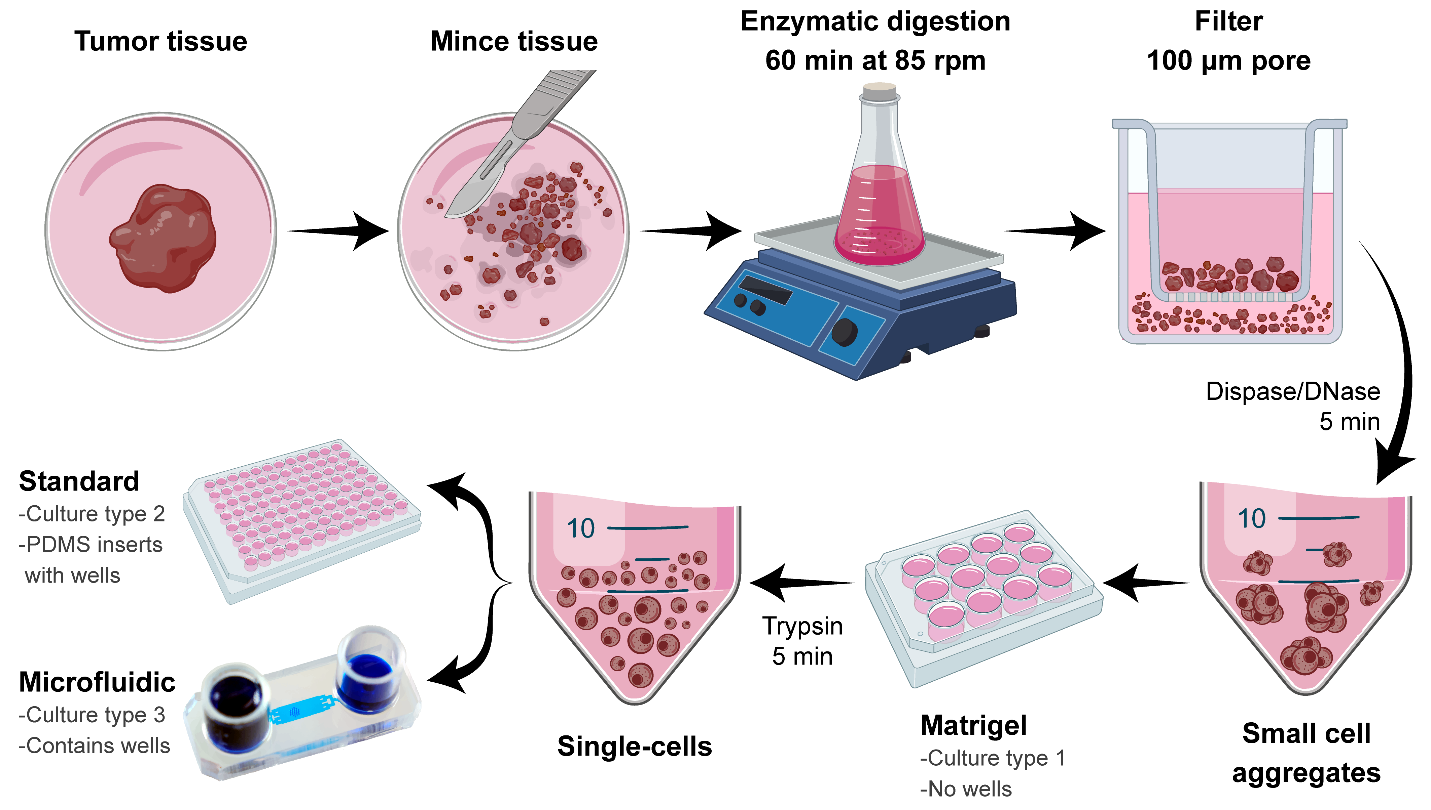


**Figure S7.** **Workflow for ovarian cancer organoid cultures**. Schematic illustrating PDX tissue digestion followed after excision from a mouse. After dispersion into single cell suspension, cells were seeded into three different culture formats: microfluidic devices with wells, plates with Matrigel and 96-wells plates with PDMS inserts containing 250 µm wells.


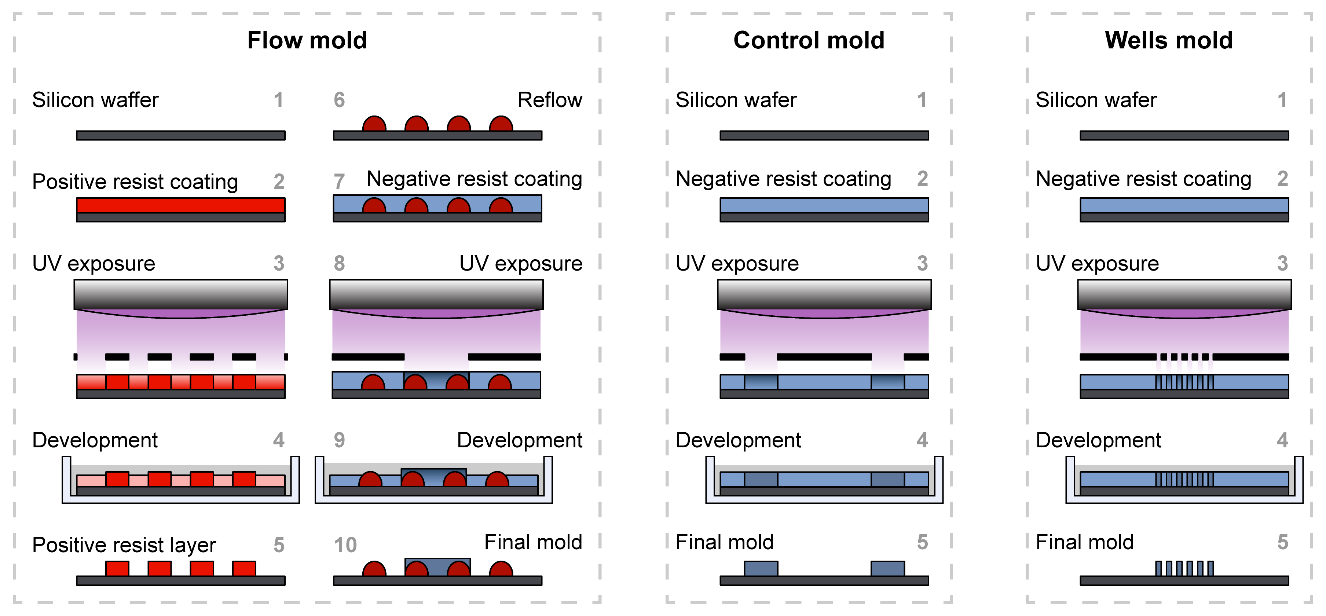


**Figure S8.** **Photolithography process – fabrication of molds.** Schematic depicting fabrication process for all three molds that comprises multi-plex device. Single-plex device was fabricated with only negative resist on flow mold and a wells mold.


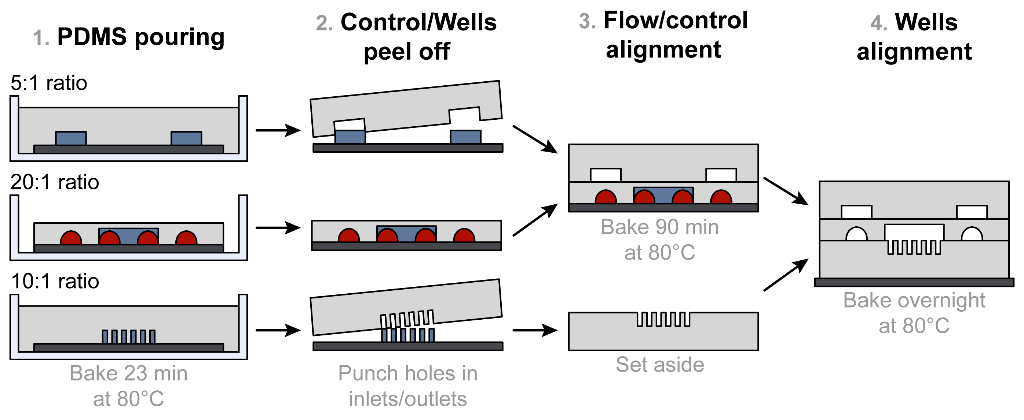


**Figure S9.** **Soft lithography – replicating molds in PDMS.** Schematic of fabrication and assembly of PDMS devices. All three PDMS layers were attached together by using different PDMS base to curing agent ratios and subsequent exposure to heat.

**Supplementary movie (available online)**

**Movie S1:** **Multi-plex device testing with food dyes.** Proof of concept of multi-plex microfluidic device operation using food dyes.
